# Métastases pleuro-pulmonaires des néoplasies extra-thoraciques

**DOI:** 10.11604/pamj.2017.26.44.10708

**Published:** 2017-01-30

**Authors:** Farid Badri, Salma Ait Batahar, Safae El Idrissi, Hafsa Sajiai, Hind Serhane, Lamyae Amro

**Affiliations:** 1 Service de Pneumologie, Laboratoire de Recherche Pneumologie-Cardiologie-Immunopathologie et Métabolisme, Université Qadi Ayyad, Marrakech, Maroc

**Keywords:** Métastase, plèvre, poumon, Metastasis, pleura, lung

## Abstract

Les poumons reçoivent la totalité du drainage veineux du corps expliquant la grande fréquence des métastases pleuro-pulmonaires de plusieurs cancers. L'objectif était d'étudier les manifestations radio-cliniques des métastases pleuro-pulmonaires des cancers extra-thoraciques. Nous rapportons une étude rétrospective de patients porteurs de métastase pleuro-pulmonaire colligés dans notre service entre janvier 2006 et décembre 2014. 76 dossiers ont été étudiés. La moyenne d'âge était de 50 ans (extrêmes allant de 21 ans jusqu'à 89 ans) avec une prédominance masculine dans 57,8% des cas. La symptomatologie clinique était faite principalement de toux (32,8% des cas), de dyspnée (23,7% des cas) et d'hémoptysie (11,2% des cas). Les cancers primitifs à l'origine des différentes métastases pleuro-pulmonaires retrouvés dans notre série sont dominés respectivement par le cancer du sein dans 27,6% des cas, les cancers digestifs dans 15,8% des cas, les cancers génitaux dans 9,2% des cas, les sarcomes dans 7,8% des cas, le cancer rénal dans 5,2% des cas, le cancer de la vessie dans 5,2% des cas, le cancer de la prostate dans 3,9% cas, les cancers ORL dans 3,9% des cas, les cancers thyroïdiens dans 3,9% des cas, le cancer de la peau dans 2,6% des cas et le cancer primitif d'origine indéterminée dans 14,4% des cas. Plusieurs aspects radiologiques des métastases pleuro-pulmonaires ont été retrouvés dans notre série, ils peuvent être isolés ou en association. L'aspect radiologique le plus fréquent est celui du lâcher de ballon présent dans 52,6% des cas, suivi des pleurésies présentes dans 34,2% des cas, des micronodules diffus présents dans 23,6% des cas et un nodule unique présent dans 3,94% des cas. Les cancers secondaires pleuro-pulmonaires sont fréquents. Ils viennent en 3^ème^ position après les métastases ganglionnaires et hépatiques et ils sont retrouvés dans 30% des autopsies de patients porteurs d'une néoplasie.

## Abstract (en)

The lungs receive the entire venous drainage of the body. This explains the high incidence of pleuropulmonary metastases originating from several cancers. The goal of this is to study the clinical manifestations of pleuro-pulmonary metastases originating from extra-thoracic cancers. We conducted a retrospective study of patients with pleuro-pulmonary metastasis whose data were collected in our department between January 2006 and december 2014. 76 patient medical records were studied. The average age was 50 years (aged 21-89 years) with a male predominance in 57.8% of cases. Clinical symptoms were mainly cough (32.8% of cases), dyspnea (23.7% of cases) and hemoptysis (11.2%). Primary cancers responsible for various pleuro-pulmonary metastases found in our case series were dominated by breast cancers in 27.6% of cases, gastro-intestinal cancers in 15.8% of cases, genital cancers in 9, 2% of cases, sarcomas in 7.8% of cases, renal cancers in 5.2% of cases, bladder cancers in 5.2% of cases, prostate cancers in 3.9% cases, ENT cancers in 3.9% of cases, thyroid cancers in 3.9% of cases, skin cancers in 2.6% of cases and cancers of unknown primary origin in 14.4% of cases respectively. Several radiologic features of pleuro-pulmonary metastases have been found in our case series; they can be isolated or combined. The most common radiologic aspect was multiple pulmonary nodules in 52.6% of cases, followed by pleurisies in 34.2% of cases, diffuse micronodules in 23.6% of cases and a solitary nodule in 3.94% of cases. Secondary pleuropulmonary cancers are frequent. They come in 3rd place after lymph nodes and liver metastases and are found in 30% of autopsies of patients with neoplasia.

## Introduction

La circulation pulmonaire reçoit la totalité du sang veineux du corps humain. Ceci explique la grande fréquence des métastases pleuropulmonaires de plusieurs cancers. Celles-ci ont une prévalence de 30 à 50% chez les patients porteurs de néoplasie qu'elle soit thoracique ou extra-thoracique. Le rôle de filtre vasculaire joué par le parenchyme pulmonaire est la principale explication des métastases pulmonaires. Les néoplasies extra-thoraciques les plus pourvoyeuses de métastases pulmonaires et pleurales sont les cancers du sein, du colon, du pancréas, des reins, de l'estomac, de la sphère oto-rhino-laryngologique (ORL) et les mélanomes. Cette étude a été réalisée à fin de déterminer les manifestations cliniques et radiologiques des métastases pleuropulmonaires des cancers extra-thoraciques.

## Méthodes

Il s'agit d'une étude rétrospective portant sur 76 cas de patients porteurs de métastase pleuro-pulmonaire colligés dans notre service entre janvier 2006 et décembre 2014.

## Résultats

**Figure 1:**
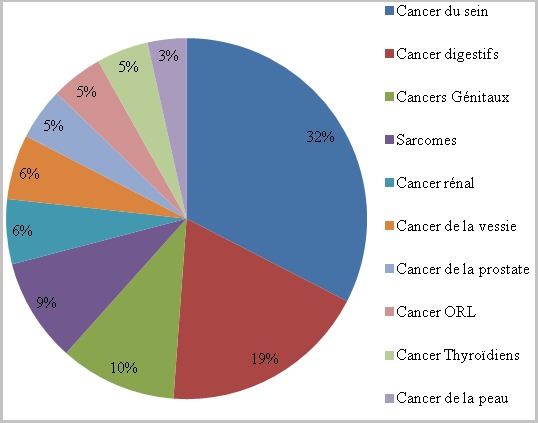
Coupes tomodensitométriques en fenêtre parenchymateuse pulmonaire. Métastases pulmonaires excavées d’un cancer ovarien chez une patiente de 45ans

**Figure 2:**
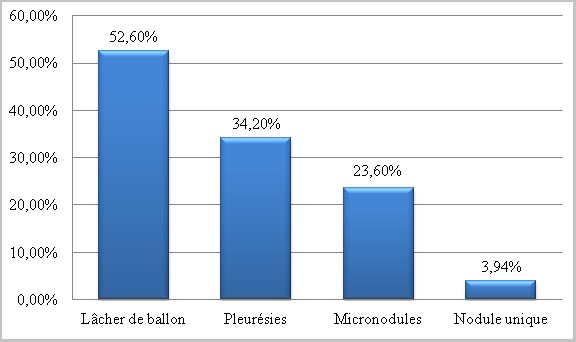
Répartition des cancers à l’origine de métastases pleuropulmonaires

**Figure 3:**
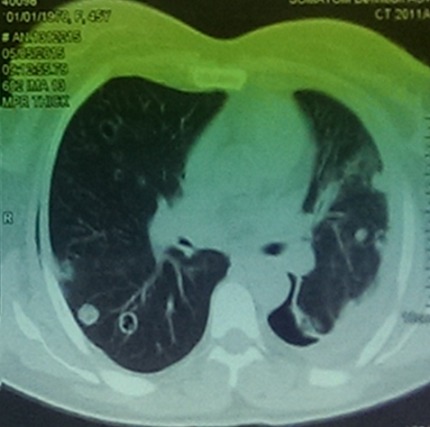
L’aspect radiologique des métastases pleuropulmonaires

La moyenne d'âge était de 50 ans (extrêmes allant de 21ans jusqu'à 89 ans) avec une prédominance masculine dans 57,8% des cas. La symptomatologie clinique était principalement faite de: toux dans 32,8% des cas, dyspnée dans 23,7% des cas et hémoptysie dans 11,2% des cas. Les cancers primitifs à l'origine des différentes métastases pleuropulmonaires retrouvés dans notre série sont dominés respectivement par le cancer du sein dans 27,6% des cas, les cancers digestifs dans 15,8% des cas, les cancers génitaux dans 9,2% des cas (Figure 1), les sarcomes dans 7,8% des cas, le cancer rénal dans 5,2% des cas, le cancer de la vessie dans 5,2% des cas, le cancer de la prostate dans 3,9% cas, les cancers ORL dans 3,9% des cas, les cancers thyroïdiens dans 3,9% des cas, le cancer de la peau dans 2,6% des cas et le cancer primitif d'origine indéterminée dans 14,4% des cas (Figure 2). Différents aspects radiologiques, isolés ou en association, ont été retrouvés dans notre série. A l'imagerie thoracique, l'aspect le plus retrouvé est celui de multiples opacités de tailles différentes diffuses aux deux champs pulmonaires réalisant l'image en lâcher de ballon. Cet aspect était présent dans 52,6% des cas, suivi des pleurésies qui étaient présentes dans 34,2% des cas. Un syndrome interstitiel fait de micronodules diffus a été retrouvé dans 23,6% des cas et un nodule unique a été retrouvé dans 3,94% des cas (Figure 3).

## Discussion

### Modes de dissémination tumorale

*Extension directe:* la dissémination tumorale par extension directe représente un mode de dissémination tumorale beaucoup plus rare que l'atteinte par diffusion hématogène ou lymphatique. Le plus souvent, Il s'agit de tumeurs primitives thyroïdiennes, thymiques ou œsophagiennes. La rupture capsulaire de ganglions tumoraux intramédiastinaux peut entrainer une extension directe à la trachée ou aux poumons.

### Diffusion à distance

*Type d'atteinte:* la progression d'un processus tumoral résulte volontiers d'une dissémination en cascade avec atteinte successive de plusieurs organes relais [1]. Après le relais ganglionnaire, le poumon est l'organe le plus fréquemment atteint, avant le foie, l'os et le cerveau [2]. L'atteinte pulmonaire se présente de trois manières différentes [1, 3].

**Filtre pulmonaire initial et exclusif:** ce mode de dissémination intéresse les carcinomes de la tête et du cou, les tumeurs du rein, du testicule, les choriocarcinomes, les sarcomes ostéogéniques et les sarcomes des tissus mous, les mélanomes, et enfin certaines tumeurs endocrines, en particulier les carcinomes thyroïdiens et surrénaliens.

**Atteinte pulmonaire séquentielle:** les MP surviennent souvent après les métastases hépatiques puisque le foie est le premier filtre des tumeurs du tractus gastro-intestinal, et parfois de l'ovaire. les métastases osseuses peuvent survenir en premier en cas de tumeurs prostatiques. Les cancers de l'œsophage, les cancers rectaux mais aussi coliques, théoriquement drainés par le système porte, peuvent néanmoins générer des MP isolées sans métastase hépatique.

**Atteintes métastatiques simultanées:** le poumon, le foie et les os sont atteints de façon simultanée au cours de néoplasies de la vessie, de l'utérus (corps ou col) et du sein qui métastasent au foie et au poumon indépendamment par voie lymphatique et veineuse systémique.

**Voies de dissémination:** les voies de dissémination qu'emprunte le matériel cellulaire néoplasique pour le développement des métastases pulmonaires sont au nombre de cinq [1], bien qu'il existe fréquemment une association des différentes modalités de dissémination chez un même patient.

### Métastases pulmonaires hématogènes

*Artères pulmonaires:* la voie de dissémination hématogène, la plus fréquente, est caractérisée par le passage de l'embole cellulaire néoplasique issu de la tumeur initiale dans les veines systémiques.

**Artères bronchiques:** ce mécanisme de dissémination est beaucoup plus rare que le précédent. Il explique peut-être le développement de certaines métastases endobronchiques [4].

**Lymphatiques intrapulmonaires et pleuraux** [1, 5]: l'extension néoplasique intrapulmonaire se fait le long et au sein des vaisseaux lymphatiques contenus dans l'interstitium péribronchovasculaire, les septa interlobulaires et la plèvre.

**Espace pleural:** le développement du foyer pleural initial pourrait s'effectuer à partir d'une extension tumorale directe par contiguïté, en particulier en cas de néoplasie mammaire ou pulmonaire ou d'une extension via les lymphatiques ou les vaisseaux sanguins.

**Voies aériennes:** c'est une voie de dissémination moins importante qui explique surtout l'atteinte bilatérale multifocale du cancer pulmonaire primitif bronchioloalvéolaire, et certaines métastases pulmonaires des cancers pharyngolaryngés.

**Aspects radiologiques des métastases pulmonaires:** les métastases pulmonaires peuvent prendre plusieurs aspects radiologiques qui peuvent résulter d'un ou plusieurs mécanismes de dissémination. Plusieurs aspects radiologiques ont été retrouvés dans notre série, ils peuvent être isolés ou en association.

**Nodule pulmonaire:** c'est l'aspect le plus fréquent des MP hématogènes [1, 3] ; il peut s'agir d'un aspect de micronodules disséminés ou à l'inverse un aspect de macronodules arrondis à contours nets répartis de façon aléatoire dans un ou les deux champs pulmonaires réalisant un aspect de «lâcher de ballons» ou de miliaire carcinomateuse. La lymphangite carcinomateuse correspond à une diffusion par voie lymphatique souvent associée à une dissémination hématogène. Elle peut d'ailleurs s'associer aux nodules. Elle est observée dans 35 à 55 % des cas selon les séries autoptiques [6]. Dans notre série un aspect de «lâcher de ballons» a été retrouvé dans 52,60% des cas et un aspect de micronodules a été observé dans 23,60% des cas. Seuls 2 à 10% des nodules pulmonaires uniques sont des métastases. Certaines lésions primitives prédisposent plus que d'autres à cette situation. Il s'agit surtout d'adénocarcinomes de la charnière rectosigmoïdienne, de sarcomes osseux, de carcinomes du rein, du sein, de tumeurs testiculaires et de mélanomes cutanés [4]. Dans notre série on notre un aspect de nodule pulmonaire unique dans 3,94% des cas.

**Métastases pleurales:** elles sont les tumeurs malignes de la plèvre les plus fréquentes, représentant 95% des cas environ. Il s'agit le plus souvent d'adénocarcinomes d'origine pulmonaire, mammaire, ovarienne et gastrique, les lymphomes pouvant également envahir la plèvre. La cause la plus fréquente d'épanchements pleuraux malins est un cancer pulmonaire sous-jacent (36%). Ce mode de présentation survient chez 15% des patients lors du bilan initial et chez 50% d'entre eux en cas de maladie disséminée. Le carcinome mammaire est la seconde cause d'épanchement malin (25%), suivi par les lymphomes (10%) et les carcinomes gastriques et ovariens (5% ou moins). Chez environ 10% des patients qui présentent un épanchement pleural malin, le site primitif n'est pas retrouvé [7]. Dans notre série 34,20% des cas avait une pleurésie métastatique d'un cancer extrathoracique. D'autres aspects radiologiques des métastases pulmonaires ont été décrits dans la littérature mais non rapportés dans notre série, à savoir les embolies tumorales et les métastases endobronchiques.

### Métastases pulmonaires selon les principales localisations primitives

Toutes les localisations néoplasiques peuvent atteindre les poumons au cours de leur diffusion métastatique. Les métastases pleuro-pulmonaires (MPP) ont une prévalence de 30 à 50% chez les patients porteurs de néoplasie thoracique ou extrathoracique [8]. Dans un quart des cas environ, le poumon est le seul site métastatique. Les métastases pulmonaires des cancers du sein sont fréquemment retrouvées dans la littérature et surviennent essentiellement par voie hématogène ou lymphatique. Elles représentent 20 à 35% des séries et elles sont estimées à 27,6% des cas rapportés dans notre étude [9]. Les cancers digestifs viennent au deuxième plan dans notre série dans 15,8% des cas, ce qui rejoint les résultats retrouvés dans d'autres séries qui parlent de 15% à 20% des cas [10]. Il s'agit notamment des cancers de: l'œsophage, l'estomac, le colon et le rectum. Le poumon est le premier site métastatique des cancers du rein, avec une incidence de 55 à 77% dans les séries autopsiques [11]. Dans notre série, Les métastases pulmonaires du cancer du rein/vessie représentent 10,4% des cas en comparaison avec d'autres séries (10 à 12% des cas) rapportées par la littérature. L'atteinte pulmonaire des cancers du corps utérin diffère selon l'histologie initiale, de type adénocarcinome ou sarcome. Dans une série de 817 patientes traitées, l'incidence des métastases pulmonaires était de 6% [12] avec une atteinte pulmonaire plus fréquente en cas d'adénocarcinome. Dans notre série, on note la fréquence du cancer du col et de l'utérus dans de 9,2% des cas. Les sarcomes et en particulier les ostéosarcomes se compliquent fréquemment d'une atteinte pulmonaire. Cette atteinte est précoce au cours de leur évolution, jusqu'à 20 à 30% dans les séries récentes [13] avec une atteinte pulmonaire initiale de 7 à 8% [14]. Sur une étude rétrospective de 108 dossiers d'ostéosarcome, 30 cas de métastase pulmonaire soit 27,7% ont été retrouvés. Dans notre série on a rapporté 7,8% de métastase pulmonaire. La fréquence des métastases pulmonaires au cours des cancers ORL varie en fonction de la localisation initiale. Elle est de 10% en cas d'atteinte oropharyngée et peut atteindre 60% en cas d'atteinte laryngée [15]. Dans notre série, 3,8% des cas qui ont développé une métastase pulmonaire. L'atteinte pulmonaire au cours du carcinome anaplasique de la thyroïde est fréquente mais reste secondaire, le pronostic étant lié à l'extension locale et à l'obstruction trachéale qui peut être très rapide. Dans notre étude, 3,9% des cas ont développés des MPP d'origine thyroïdienne. Le séminome testiculaire atteint souvent le thorax avec une fréquence de l'ordre de 15% lors du diagnostic initial. Une fréquence de 3,9% des cas a été notée dans notre série. L'atteinte pulmonaire au cours de l'évolution du mélanome est peu fréquente, avec une prévalence de 12% [16]. Dans notre série, on note une prévalence de 2,6%. Le cancer primitif à l'origine des métastases pleuro-pulmonaires reste parfois difficile à déterminer, ainsi 15% des cas dans notre série ont été répertoriés. Les problèmes diagnostiques qui se posent devant des métastases pleuro-pulmonaires sont l'identification des métastases et leur différenciation des autres pathologies et la recherche du cancer primitif à chaque fois que celui-ci n'est pas connu.

## Conclusion

Les cancers secondaires pleuro-pulmonaires sont fréquents. Ils viennent en 3^ème^ position après les métastases ganglionnaires et hépatiques et ils sont retrouvés dans 30% des autopsies de patients porteurs d'une néoplasie.

### Etat des connaissances actuelles sur le sujet

- La fréquence des métastases pleuro-pulmonaires des néoplasies extra-thoraciques;
- La dissémination pleuro-pulmonaire des néoplasies extra-thoracique se fait le plus souvent par diffusion hématogène ou lymphatique, alors que l'extension directe représente un mode de dissémination tumorale beaucoup plus rare;
- Les métastases pleuro-pulmonaires des cancers du sein sont les plus fréquemment retrouvées.

### Contribution de notre étude à la connaissance

- Données sur la fréquence des métastases pleuro-pulmonaires des néoplasies extra-thoraciques dans le contexte marocain;
- La symptomatologie respiratoire peut être révélatrice de plusieurs cancers extra-thoracique;
- Les images en lâcher de ballon doivent faire évoquer en premier des métastases pulmonaires. La recherche du cancer primitif doit se faire à chaque fois que celui-ci n'est pas connu.
